# Immunochemical, biomolecular and biochemical characterization of bovine epithelial intestinal primocultures

**DOI:** 10.1186/1471-2121-6-42

**Published:** 2005-12-01

**Authors:** Dorina Rusu, Suzanne Loret, Olivier Peulen, Jacques Mainil, Guy Dandrifosse

**Affiliations:** 1 Department of Biochemistry and General Physiology, University of Liege, Institute of Chemistry B6C, B-4000 Liege (Sart-Tilman), Belgium; 2 Department of Biology, University of Namur (FUNDP), Rue de Bruxelles, 61, B-5000 Namur, Belgium; 3 Department of Infectious and Parasitic Diseases/Bacteriology, Faculty of Veterinary Medicine, University of Liege, Boulevard de Colonster B43, B-4000 Liege (Sart-Tilman), Belgium

## Abstract

**Background:**

Cultures of enterocytes and colonocytes represent valuable tools to study growth and differentiation of epithelial cells. In vitro models may be used to evaluate passage or toxicity of drugs, interactions of enteropathogenes bacteria strains with intestinal epithelium and other physiologic or pathologic phenomenon involving the digestive tract.

**Results:**

Cultures of bovine colonocytes and jejunocytes were obtained from organoid-enriched preparations, using a combination of enzymatic and mechanical disruption of the intestine epithelium, followed by an isopicnic centrifugation discarding most single cells.

Confluent cell monolayers arising from plated organoids exhibited epithelium typical features, such as the pavement-like structure, the presence of apical microvilli and tight junctions. Accordingly, cells expressed several markers of enterocyte brush border (i.e. maltase, alkaline phosphatase and fatty acid binding protein) as well as an epithelial cytoskeleton component (cytokeratin 18). However, enterocyte primocultures were also positive for the vimentin immunostaining (mesenchyme marker). Vimentin expression studies showed that this gene is constitutively expressed in bovine enterocytes. Comparison of the vimentin expression profile with the pattern of brush border enzymes activities, suggested that the decrease of cell differentiation level observed during the enterocyte isolation procedure and early passages of the primoculture could result from a post-transcriptional de-repression of vimentin synthesis. The low differentiation level of bovine enterocytes *in vitro *could partly be counteracted adding butyrate (1–2 mM) or using a glucose-deprived culture medium.

**Conclusion:**

The present study describes several complementary approaches to characterize bovine primary cultures of intestinal cells. Cultured cells kept their morphologic and functional characteristics during several generations.

## Background

Intestinal epithelium is organized as a single layer which covers the luminal side of this part of the digestive tract. Cells that form this inner cover present specialisations according to their role in the digestive function but they have also common functions and are continuously renewed by the stem cell proliferation. *In vivo*, these stem cells, known as progenitors of all cell types, are mostly located in the lower third of the epithelium crypts, which are epithelial invaginations into the *lamina propria*. Cells differentiation is associated with their migration from the depth of the crypts to the top of the villi and is followed by the cells death and desquamation into the intestinal lumen (for review, see: [1,2]).

Enterocyte cultures represent valuable tools to assess the passage and/or toxicity of drugs, as well as the molecular mechanisms operating in pathologies caused by infectious agents known to affect the intestinal epithelium integrity (i.e. microvilli effacing microbial strains). However, the successful establishment of an intestinal cell culture is hampered by the high rate of cell death occurring when isolating them from the epithelium (for review: see [3]) and the difficulty to select proliferating cells to ensure several cell generations *in vitro*.

Although several investigators have already developed culture methods of intestinal cells for animal species such as the mouse [4-8], the rabbit [9-11] or the pig [12,13], until now only two studies reported the production of primary cultures from intestinal bovine cells. More precisely, Dibb-Fuller and coworkers [14] established a procedure for obtaining such cultures from ileum and colon. Föllmann and collaborators [15] also used the bovine colon as a source of intestinal cell cultures.

Various strategies were reported in order to isolate enterocytes from the colon or the small bowel. Among the oldest methods, the mechanical dissociation [16] provided rapidly a viable cell preparation but was frequently associated with a fibroblast contamination. The chelating methods also generated isolated epithelial cells retaining their morphologic characteristics. However, while first attempts seemed to affect cell surface receptors [17-19], further applications of chelating agents for a short time were suitable for the production of uncontaminated cultures of human colonocytes [20]. Matrisperse, a non enzymatic solution initially designed to isolate epithelial cells grown on Engelbreth-Holm-Swarm (EHS) biomatrix, also allowed the dissociation of the integral villus epithelial lining from human intestinal biopsies that produced a confluent monolayer *in vitro *[21]. The enzymatic digestion provided a cell preparation made up mainly of organoids (crypt-like cell aggregates) that were shown to successfully reconstitute either a monolayer of epithelial cells *in vitro *or crypt-villus structures *in vivo *following grafting in various tissues [15,20,22-27]. In spite of these numerous efforts, little is known about the cell differentiation evolution over culture generations.

The present study describes several complementary approaches to characterize bovine primary cultures of intestinal epithelial cells called jejunocytes and colonocytes as they were respectively isolated from adult jejunum and spiralled colon. Cultures were initiated using an organoid-enriched suspension obtained by a multi-step method. Results of vimentin expression in both types of enterocyte primary cultures are discussed in terms of differentiation status of cells.

## Results

### Morphological features of jejunocyte and colonocyte cultures

**Figure 1:**
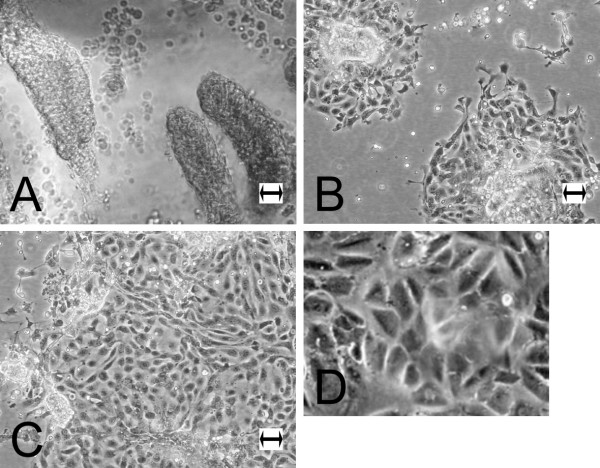
**Morphology of cell culture. **Aspect, under phase contrast microscopy, of primoculture evolution of bovine intestinal enterocytes, starting from (A) adherent organoid giving larger (B) proliferating foci that joined together into (C) confluent monolayers presenting typical (D) close-up of a confluent monolayer showing a dome. Bar: 25 μm.

The method developed to isolate cell material from the bovine jejunum and spiraled colon led to the production of suspension enriched in undissociated cell aggregates (or organoids; Figure 1A) adhering to collagen coated culture flasks. They give rise to circular proliferating foci (Figure 1B). Within 5 days the initial foci enlargement had led to the fusion of cell plaques in one confluent layer (Figure 1C). Confluent monolayers arising from the initial seeding of cultures presented a heterogeneous aspect (Figure 1C) due to the presence of residual multicellular organoids dispersed in the newly formed monolayer. With the first passage jejunocyte and colonocyte cultures acquired a homogeneous pavement-like aspect typical of epithelial sheets. We generally carried out 7 to 9 passages (with split ratio 1:2 or 1:3) over 4 to 5 weeks in both cell series (jejunocytes and colonocytes) without major changes of cell morphology. During early passages (1–3), the confluence recovery was achieved within 2 to 3 days and then a progressive slowdown in the cell proliferation rate was observed until cessation. Confluent monolayers of both cell types exhibited domes, appearing as round blurred arrays corresponding to fluid entrapment between the flask wall and the basolateral side of the monolayer, resulting from ionic regulations by functional epithelial cells (Figure 1D). This observation is in agreement with dome formation in cultures of the human intestinal cell lineage Caco-2 [28].

**Figure 2:**
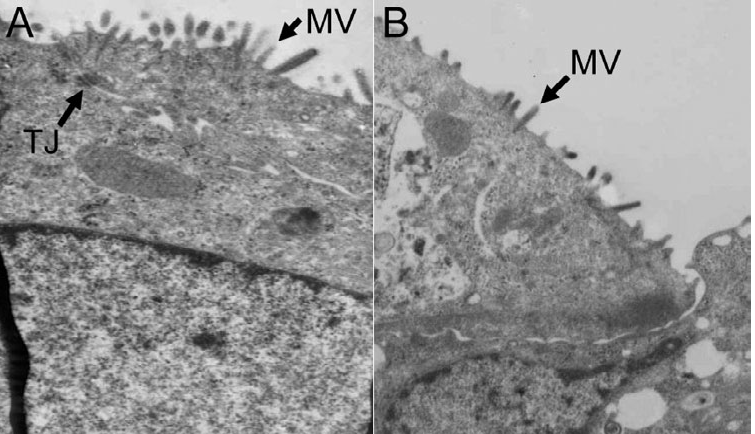
**Ultrastructure of jejunocytes and colonocytes. **Electron micrographs of monolayer cultures of bovine jejunocytes (A) and colonocytes (B). Note the presence of microvilli (MV) and tight junctions (TJ). Magnification: 6,000 ×.

Ultrastructural analyses of cultured cells revealed apical tight junctions (Figure 2A) between cells forming the monolayer of both cell types. Cultured jejunocytes and colonocytes also exhibited a few apical microvilli (Figure 2A, B).

To ascertain the identity and functionality of cultured monolayers, cells components from various culture passages were submitted to immunolabelling (immunocytochemistry and Western blot). These data were compared to results from gene expression studies (RT-PCR) and measurements of specific activities of brush border associated enzymes.

### Cell characterization using immunodetection

**Figure 3:**
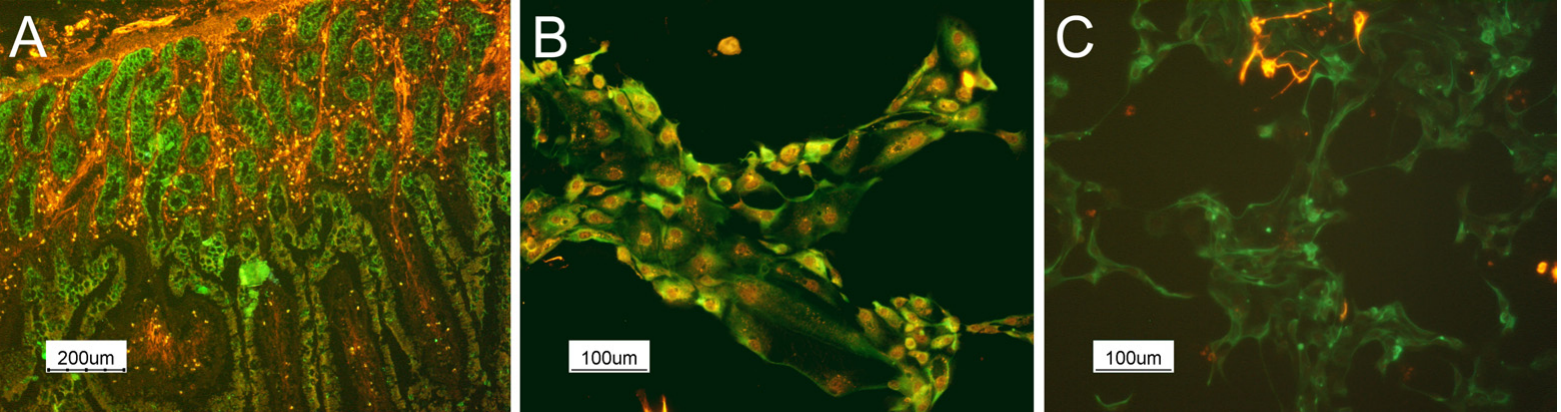
**Immunostaining of cryosections and primocultures. **Double-immunostainings: (A) pan-cytokeratin (FITC conjugate) and vimentin (revealed by a Cy3 conjugate) in small intestine cryosections; and (B) in enterocyte primocultures; (C) pan-cytokeratin (FITC conjugate) and alpha actin (revealed by a TRITC conjugate) in of bovine enterocyte primocultures.

**Figure 4:**
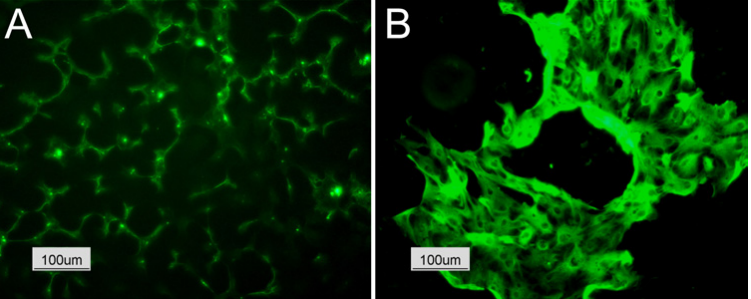
**Immunostaining of bovine enterocytes. **Indirect immunofluorescence of (A) the epithelium specific antigen and (B) of cytokeratin 18 in bovine enterocytes.

Antibodies directed against cytokeratins (intermediary type II filaments specifically expressed in epithelia) and vimentin (intermediary type III filaments) were used to distinguish cells of epithelial origin from contaminating fibroblasts. Double immunolabeling of cryosections of the bowel wall showed the presence of the cytokeratin positive cells all over the lining epithelium and vimentin positive cells in the submucosa (Figure 3A). Cytokeratins showing the characteristic disposition of intermediate filaments were also recognized in obtained monolayers. Surprisingly, double staining vimentin-cytokeratin (Figure 3B) clearly showed that cultured cells expressed both epithelial and mesenchymal markers. However, the double staining cytokeratin-α-actin (Figure 3C) distinguished separate cells populations. The few α-actin positive cells, probably contaminant myofibroblasts (and/or smooth muscle cells) were released from the gut wall during the dissociation procedure and not completely eliminated by the sorbitol centrifugation or the selective attachment. Indirect immunofluorescence revealed also the membrane distribution of the epithelial specific antigen (ESA, a cell surface glycoprotein) in cultured enterocytes (Figure 4A) and intracellular distribution of the cytokeratin 18 (Figure 4B). E-Cadherin representing one of the proteins located at the adherence junctions was also detected, but a mis-localization was observed. The cells did not present the characteristic staining pattern to the cell periphery, but a granular distribution into the cytoplasm (data not shown).

**Figure 5:**
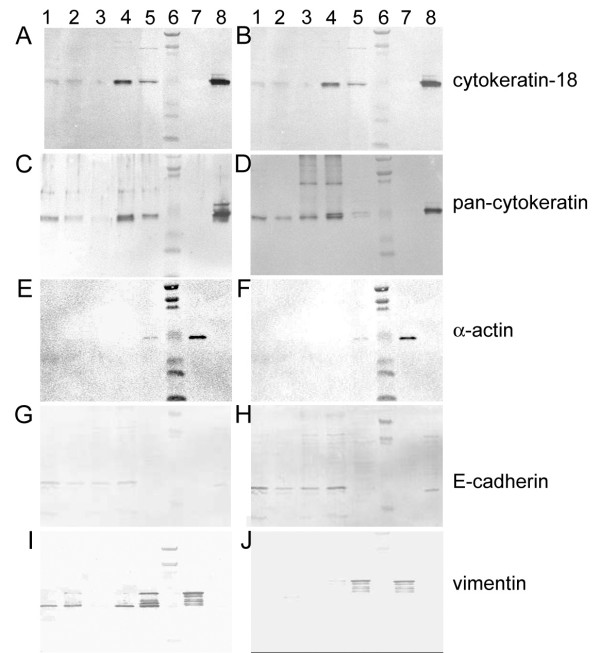
**Western blot of cell markers in jejunum, colon, jejunocyte and colonocyte proteins. **Immunodetection of cytokeratin-18 (A, B), others acidic and basic cytokeratins by an anti-pan cytokeratin antibody (C, D), α-actin 1A4 (E, F) and E-cadherin (G, H) and vimentin (I, J) in cellular extracts separated by SDS-PAGE (10%) and transferred on PVDF membranes. A, C, E, I were jejunum and jejunocyte samples. B, D, F, J were colon and colonocyte samples. Lanes: 1, freshly scrapped epithelia; 2, organoids suspension; 3, primary culture of bovine enterocytes; 4, cultured enterocytes after the first passage; 5, cultured enterocytes after the second passage; 6, broad range protein standards molecular weight ranging from 5.7 KDa to 198 Kda; 7, cultured 3T3-fibroblast used as positive control for vimentin and α-actin staining and 8, cultured Caco-2 cells used as positive control for cytokeratin and E-cadherin staining.

Western blot analyses also confirmed the presence of epithelial characters in cultured cells. Pan-anti-cytokeratin and anti-cytokeratin 18 antibodies recognised the same major 45 KDa protein (Figure 5A, B) in homogenates from: (1) freshly scrapped intestinal epithelium, (2) organoid suspensions used to seed cultures, (3) intestinal cell cultures of both types, and in the (4) positive control Caco-2 cells. No cytokeratin staining was noticeable in 3T3 fibroblasts. The size uniformity of stained products in all tested epithelial cells was also confirmed using the pan-cytokeratin antibody (major signal at 45 KDa, Figure 5C, D). In addition, blots were also positive for another epithelial cell marker, the E-cadherin (around 35 KDa; Figure 5G, H). Compared to the expected size (120 KDa), the anti-E cadherin target likely presented the size of a protein domain (35 KDa, Figure 5H). The antibody used was directed against the intracellular domain of this protein.

In agreement with immunocytochemistry results, vimentin and α-actin presented distinct distribution in sample homogenates. Indeed, the anti-vimentin targeted the same proteins in all bovine intestinal cell cultures and in the control 3T3 fibroblasts (Figure 5I, J), while α-actin was scarcely detected in more advanced passages (Figure 5E, F). Taken together, western blot and immunocytochemistry results strongly suggested that vimentin is expressed in primary cultures of epithelial intestinal cells from bovine jejunum and colon. However, vimentin was not detected by western-blot in Caco-2 cell proteins (Figure 5I, J). Although immunocytochemistry results for α-actin indicated a very low contamination of bovine epithelial cell cultures by mesenchymal cells, western blot results indicated that this phenomenon is to low to be detected in most culture stages.

### Gene expression analyses of enterocyte markers

**Figure 6:**
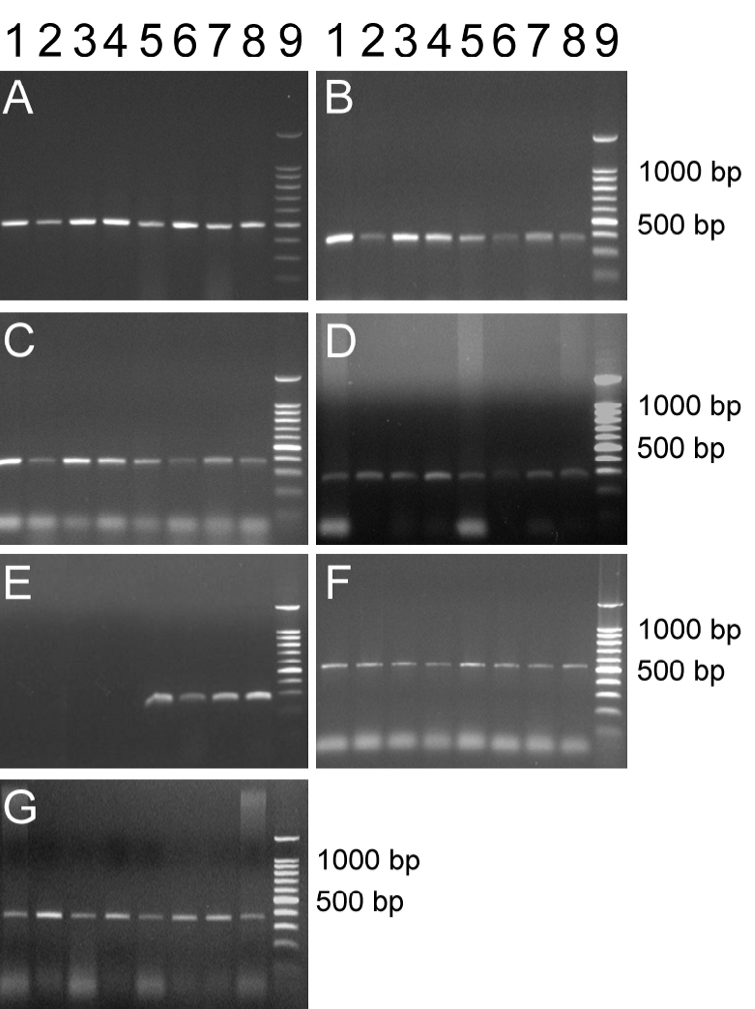
**Gene expession analysis in bovine intestinal epithelium, organoids and cultured cells. **RT-PCR analysis of several gene expression in bovine intestinal epithelia, isolated organoids and cultured cells. The fragment size (bp) of amplified cDNA fragment was determined after migration on agarose (1%) gels and subsequent staining with ethidium bromide. Primers directed against (A) β-actin, amplicon size 485 bp, used as a quality control of cDNA obtained by reverse transcription. Amplifications of epithelial and enterocyte specific markers were obtained using primers directed against (B) FABP, 568 bp, (C) villin, 384 bp, (D) ZO1, 272 bp, (E) small intestinal peptidase, 276 bp, (F) vimentin, 548 bp and (G) E-cadherin, 308 bp. Lanes: 1, freshly scrapped colon epithelium; 2, organoid suspension from colon; 3, primary culture of colonocytes; 4, cultured colonocytes after the first passage; 5, freshly scrapped jejunum epithelium; 6, organoid suspension from jejunum; 7, primary culture of jejunocytes; 8, cultured jejunocytes after the first passage; 9, molecular weight standard in bp.

RT-PCR analyses allowed us to investigate the expression of bovine enterocyte markers namely: villin (actin-caping protein in microvilli), zonula occludens (ZO1, associated with the cytoplasmic surface of tight junctions), fatty acid binding protein (FABP), small intestine peptidase (IP) and E-Cadherin. Expressions studies were also used to confirm the presence of the vimentin transcript in bovine cells. Similarly to western blot analyses, gene expressions studies were carried out on total RNA extracted from bovine samples of: (1) freshly scrapped epithelia, (2) organoid suspensions used to seed cultures and (3) intestinal cell cultures of both types. As can be seen in figure 6 (A–G), cDNA amplification products presented the expected size (see methods) using specific primers for coding regions of the epithelial markers, such as the villin gene (Figure 6C), the ZO1 gene (Figure 6D) and the E-cadherin (Figure 6G), that were uniformly expressed in cells from primary cultures of bovine enterocytes. Besides these structural components expected in any epithelium, we also investigated the expression of functional markers of digestive epithelia. Among them, the gene coding for the fatty acid binding protein (FABP, Figure 6B), presented a constant expression in both jejunocytes and colonocytes *in vitro*. The second tissue specific marker studied was the gene coding for the intestinal peptidase (IP, Figure 6E). In this case, the expression was restricted to jejunocyte samples, as logically expected on the basis of the functional specialization of the jejunum (nutrient processing and absorption). The IP expression could then be considered as suitable jejunocyte marker. In accordance with immunostaining results, samples from jejunocyte and colonocyte cultures expressed the vimentin gene (Figure 6F). Interestingly, while western blots analyses failed to reveal vimentin either in freshly removed epithelia or in organoids suspensions used to seed cultures, the transcript of vimentin gene was clearly detected in those samples, indicating that the vimentin is constitutively expressed in jejunum, as well as in colon from the bovine intestine. However, western blot results suggested that vimentin synthesis seemed to be restricted in cultured cells with a progressive increase over the culture passage number (Figure 5I, J).

### Specific activities of two brush border-associated enzymes

**Figure 7:**
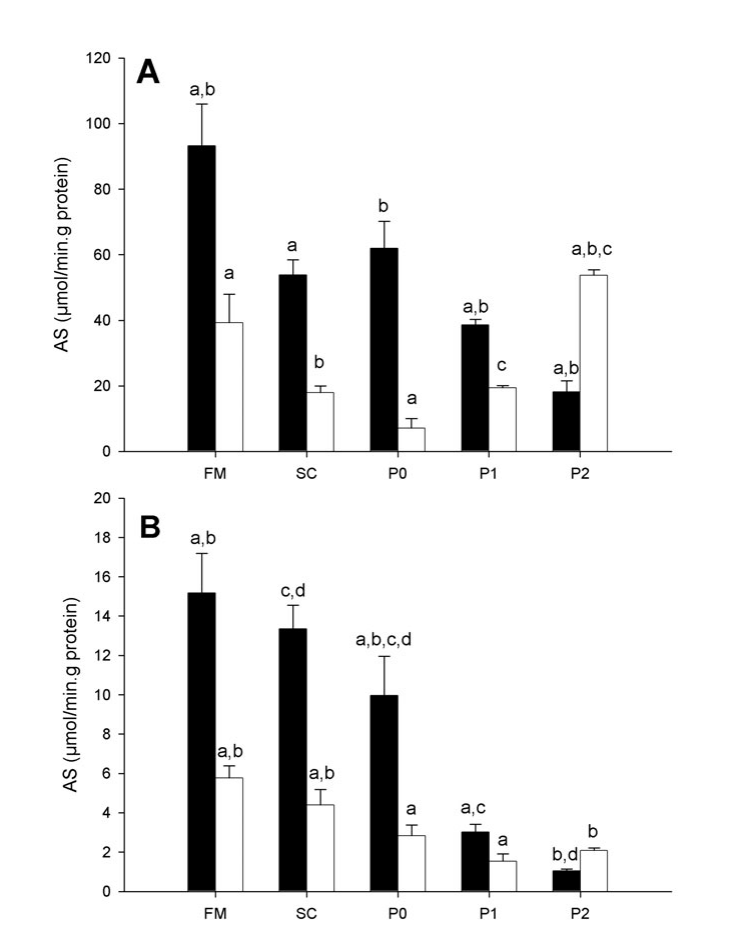
**Enzymatic characterization of fresh mucosa, seeded cells, primary culture and cultured cells. **Specific activities of (A) maltase and (B) alkaline phosphatase from cell homogenates of fresh mucosa (FM), seeded cells (CS), primary cultured cells (P0), cultured cells after the first passage (P1) and cultured cells after the second passage (P2). Homogenates were made at confluence of the monolayer. Black boxes represent jejunum cells and open boxes colon cells. Results are expressed as mean values ± SD for three culture samples. Inside the same organ group, boxes sharing the same supercript letter are significantly different (p < 0.05, N = 3).

The chosen enzymes were a disaccharidase (maltase) and the alkaline phosphatase. As for gene expression studies, enzyme activities were investigated in bovine samples of: (1) freshly scrapped epithelia, (2) organoid suspensions used to seed cultures and (3) intestinal cell cultures of both types. Assays performed on fresh epithelial tissue confirmed that jejunum and colon differed in respect to enzyme specific activities (SA), the jejunum presenting the highest level. Maltase SA (i.e. differentiation marker) clearly decreased in primary cultures of both cells types (Figure 7A).

Furthermore, the cell isolation procedure selected disaccharidase depleted material, since organoid suspensions presented a 50% reduction in regard to the fresh epithelium preparation. It is interesting to note that, compared to the organoids suspension, jejunocytes from the first culture passage did not present a drastic reduction of the maltase SA. However, with subsequent culture passages the decreasing of disaccharidase SA was progressing toward a stable low level. Similar results were obtained from measurements of the intestinal alkaline phosphatase SA (IAP, Figure 7B), a ubiquitous enzyme that is a marker of brush border in intestinal epithelial cultures. Indeed, as expected from functional differences between jejunum and colon *in vivo*, epithelia homogenates, organoids suspensions, and cultures at first passages from bovine jejunum presented higher SA for this enzyme. Moreover, similarly to what was noted for maltase SA, IAP presented a strong decrease from fresh tissue to subcultured cells, so that jejunocyte and colonocyte SA values joined together to the same low level as the culture passage number increased.

**Figure 8:**
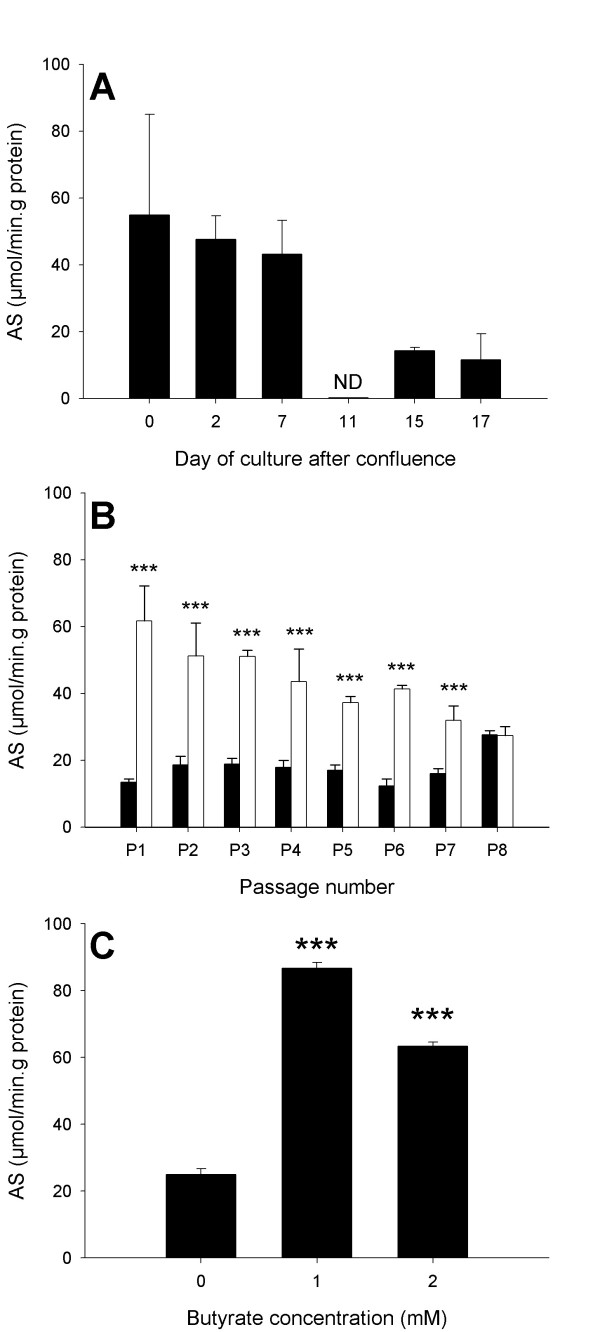
**Enzymatic characterization of confluent cultures. **Specific activity of maltase (A) over the age of a confluent culture of jejunocytes at the first passage, (B) over the increasing passage number of confluent cultures of jejunocytes (open boxes) compared to the same culture during 7-days in a inosin-containing glucose-deprived culture medium (black boxes) or (C) maintained 7 days in a medium added of sodium butyrate (1–2 mM). Results are expressed as mean values ± SD for three culture samples. ND =not detected, ***p < 0.001.

Regarding a given passage number, the maltase SA decreased over the culture duration (Figure 8A). These results reflected a loss of cell differentiation *in vitro *compared to the *in vivo *level. Adjustments of cell culture medium were made to improve the differentiation status of jejunocytes and colonocytes *in vitro*. To this end the glucose was substituted by inosin in the culture medium; this condition was described to stimulate the acquisition of the enterocyte differentiated phenotype *in vitro *[29]. As seen in figure 8B, the use of a inosin-containing glucose-free culture medium during 7 days, led to an increase of maltase activity. However this effect diminished as the passage number increased, suggesting that glucose substitution by inosin should be complemented by other culture medium modifications. Addition of sodium butyrate (1–2 mM), a substance thought to promote enterocyte differentiation through a stimulation of the CDX2 homeobox gene expression [30], proved to be efficient to stimulate maltase SA (Figure 8C).

## Discussion

Morphological data presented in the present study indicated that primary cultures of bovine enterocytes isolated from colon and jejunum presented characteristics of epithelial cells, such as a typical pavement-like aspect, the formation of domes and apical tight junctions and microvilli in confluent cultures. These bovine intestinal cells were shown to express *in vitro *epithelial cell markers such as brush border enzymes (maltase and alkaline phosphatase) and the epithelium typical cytoskeleton proteins, the cytokeratins. The enterocyte isolation procedure developed in the present study proved efficient to avoid a noticeable contamination from α-actin-positive cells (presumably myofibroblasts) in the two first steps of each type of culture (initial primary culture and first passage). The first passage also corresponded to cells presenting still a substantial differentiation level (in terms of brush border enzyme activities). To this respect, the first passage of each type of cultures appeared to be suitable to establish immortalized cell lines, a task which is now underway in our laboratory.

The functional differentiation state of the colon cells in culture could be estimated by the activities of drug metabolizing enzymes [31]. Indeed, bovine colon epithelial cell culture were characterized, as freshly isolated cells, by cytochrome P450 1A1-associated 7-ethoxyresorufin O-deethylase activity as well as by prostaglandin H-synthase-mediated production of prostaglandin E2. Activities of phase II enzymes (i.e. N-acetyltransferase 1) were also observed in colon epithelial cell cultures.

By contrast to most reports about mammalian enterocyte primocultures, data accumulated in the present study using (1) cell immunocytochemistry, (2) western blot and (3) gene expression analyses, showed that intestinal cell cultures from bovine jejunum and colon co-expressed epithelial markers and vimentin, an embryonic cytoskeleton filament that is expressed only in mesenchymal cells after birth. A second fibroblast marker, the α-actin was scarcely detected, indicating that the vimentin strong expression of culture samples did not reflect a culture contamination by mesenchymal cells. In addition, the vimentin distribution pattern did not exactly fit with the gene expression of this protein. Indeed, the immunodetection was negative in homogenates of fresh epithelia from jejunum and colon, as well as in organoid suspension used to seed each culture type, while the protein was essentially detected in samples of culture after the second passage. By contrast, the vimentin gene transcript appeared in all bovine samples analysed, including the undissociated epithelium. It seemed then that a post-transcriptional basal inhibition of vimentin synthesis has been suppressed *in vitro*. In agreement with this hypothesis, a previous research dedicated to the development of an intestinal lineage from the porcine intestine [12] led to the production of vimentin-positive cultures. In view of this result, authors postulated that cultured cells had undergone a "mesenchymal transformation" *in vitro*. Similarly, a re-expression of vimentin has been reported in epithelial cells under pathological conditions *in vivo*, as well as in primary culture [32]. Vimentin re-expression *in vitro *could be instrumental in the maintenance of cell structure and/or functions of specific proteins such as the ones associated to membrane lipid rafts [33].

Besides the possible mesenchymal transformation of epithelial cells *in vitro*, accumulating data are now in favour of a natural incidence of vimentin in the undissociated intestine epithelium. For instance, specialized M-cells found in the epithelium covering the intestine Peyer's patches were identified, among other criteria, by vimentin immunostaining [34-39]. These "Microfold-cells" are almost devoid of microvilli at their apical side as they are specialized in the transport of particulate antigens from the gut lumen to the underlying lymphoid tissue, where specific immune responses could take place. Although the structure and functions of these cells seem to be broadly admitted it is still unclear whether epithelial cells leaving the epithelium crypts are predetermined as M-cells or whether their particular phenotype developed from differentiated enterocytes at the lymphoid tissue vicinity. In favour of a possible local induction of M-cells differentiation, *in vitro *experiments using Caco-2 cells showed that the enterocyte typical phenotype could be converted in a M-like phenotype adding B lymphocytes to the basolateral side of the epithelium [40-42]. Regardless the aforementioned debate concerning the M-cells differentiation, enterocyte-lymphocyte co-culture experiments clearly demonstrated that a non-bacterial environmental factor could cause the brush border effacement of a vimentin-negative enterocyte (Caco-2 cells) leading to the phenotype of an intestinal cell type with a vimentin synthesis potential (M-like cell). Supporting the role of lymphocytes as phenotype conversion inducers, vimentin-positive cells were observed scattered throughout the villus epithelium of the rabbit small intestine, with the protein location extending from the perinuclear region to the cell membrane touching intraepithelial lymphocytes [43]. Additional indications that vimentin could be a marker of "differentiation variants" of enterocytes, came from a recent study that pointed out, in the ordinary epithelium villi of the rabbit ileum, a vimentin-positive enterocyte type sharing M-cell morphological features (brush border poor cells), but most probably representing a distinct cell type. Indeed, these so-called "cup-cells", differed from M-cells at two levels: they bound distinctive lectins and they did not take up microbeads instilled in the ileal lumen [44].

## Conclusion

Altogether, M-cells and Cup-cells features *in vivo*, as well as the experimental effacement of the enterocyte brush-border *in vitro *suggest that a vimentin re-expression could be a marker of cellular dedifferentiation. In accordance with this hypothesis, primary cultures of bovine enterocytes obtained in the present study exhibited a low differentiation level. A poor differentiated level of cultured intestinal cells has already been described by several authors using intestinal biopsies from the human [23,45,46], the mouse [25,46] or the rat [47,48]. This differentiation deficiency could stem from the preferential selection of less differentiated cells (i.e. the ones belonging to the proliferating areas of epithelial crypts) by the isolation procedure. A low differentiation level *in vitro *may also be due to the suboptimal conditions of culture. Accordingly, obtaining a primary culture of fully differentiated intestinal cells from human foetal gut, Perreault and Beaulieu [21] failed to detect vimentin neither by immunocytochemistry, nor by western-blot. Further studies would then be designed to improve the differentiation level of bovine enterocytes *in vitro *and then allow us to verify if this is associated with an inhibition of vimentin synthesis. Results presented in the present study, suggest that the combination of at least two culture medium modifications would be the first step of this study: the glucose substitution by inosin and the addition of butyrate in the culture medium.

## Methods

### Cell isolation

Intestinal fragments of proximal region of jejunum and of spiralled colon from adult animals were obtained from a local slaughterhouse. Tissue removals were made in agreement with relevant local animal welfare laws, guidelines and policies. After several washes in warm (37°C) divalent ion free PBS (PBS: 13.7 mM NaCl, 0.27 mM KCl, 0.43 mM Na_2_HPO_4_, 0.14 mM KH_2_PO_4_, pH 7.4), fragments were washed in warm PBS supplemented with 1% Antibiotic-Antimycotic solution (Gibco/BRL), 2.7 mg/ml D-Glucose (Sigma), and 4 mM L-Glutamine (Gibco/BRL). Animal organs were then immersed in the same warm supplemented PBS and transported within 30 minutes to the laboratory. All following steps were performed under a laminar flux hood (Heraeus Instruments). After several washes, fragments of intestine were filled with PBS containing 1 mM 1.4-dithiothreitol (ICN Biomedicals Inc.) and, after closing their extremities, incubated for 5 minutes in a shaking bath at 37°C, to get the epithelium surface rid of mucous before performing the cell isolation procedure. Then this incubation medium was replaced by the digesting solution consisting in supplemented PBS added of collagenase (Sigma; 300 U/ml) and dispase (Gibco/BRL; 0.1 mg/ml in PBS), for 15 minutes (for jejunum) or 20 minutes (for colon) at 37°C in a shaking bath. Using again this medium, a second digestion step lasting 45 minutes (for jejunum) or 60 minutes (for colon) was carried out in the same conditions. The lumen content (bearing mostly dead cells, as demonstrated using a viability test described below) was discarded after enzymatic incubations. Then, each intestinal segment was longitudinally wide opened and the pre-digested epithelium was scraped from the digestive mucosa using a sterile scalpel blade. The resulting material was incubated in PBS containing 1 mg/ml dispase for 10 minutes whilst active pipetting movements were done to help the dissociation of epithelium fragments. Cells and organoids (i.e. cell aggregates) were pelleted by centrifugation at 140 × g for 3 minutes.

As the large amount of single cells present in the pellet is likely to include contaminant lymphocytes (initially located in basolateral spaces between epithelial cells) and fibroblasts (released from the conjunctive subepithelial layer), the following two methods were used to impoverish the preparation in those cells: the pellet was suspended in 30 ml of Dulbecco's modified Eagle's (D-MEM) medium containing 2% of sorbitol (Sigma) and centrifuged at 50 × g for 3 minutes (isopicnic centrifugation). Under these conditions, a large part of the single cells remained on the top of the sorbitol density cushion whereas organoids accumulated at the bottom of the tube. The supernatant was then discarded and the pellet was washed in sorbitol containing D-MEM and centrifuged at 50 × g for 3 minutes. The pellet wash was repeated for about 5 times, to obtain a clear supernatant. The final pellet content, not completely devoid of single cells, was then allowed to settle down on a cell culture surface, under conditions favouring the adhesion of possible residual fibroblasts (selective attachment): the medium used to suspend the pellet was D-MEM supplemented with a high amount of FBS (10%; Gibco/BRL) and the suspension was incubated for 1 hour at 37°C in a 175 cm^2 ^culture flask (Greiner Bio-One) devoid of any coating permitting the attachment of epithelial cells. Unattached material was then removed from the flask, centrifuged at 140 × g for 3 minutes and finally the pellet suspended in the medium used to culture epithelial cells in collagen coated flasks (see below).

### Cell culture and subculture

The culture medium used was high glucose D-MEM (Gibco/BRL) supplemented with 100 nM hydrocortisone (Sigma), 20 nM triiodothyronine (Sigma), 1 ng/ml Epidermal Growth Factor (Sigma), 1 μg/ml insulin (Actrapid, Novo Nordisk A/S, Denmark), 10 μg/ml Acid linoleic/Albumin (Sigma), 1% Glutamax (Gibco/BRL) and 1% Non Essential Amino-Acids (NEAA, Gibco/BRL) [49], with 1% Antibiotic-Antimycotic solution and 2% FBS (Hyclone Perbio Sciences). The organoids were seeded at a high density, in collagen I (Roche Diagnostics; 17 μg/cm^2^) coated culture flasks (to permit epithelial cell adhesion), and the first medium change was done after 20 hours. The cultures were maintained at 37°C in a humidified incubator (Heraeus Instruments) in a 5% CO_2 _atmosphere. Medium was changed every 2 or 3 days. Confluent cells were subcultured at a split ratio of 1:2. To this end, cells were detached by incubation for 3 to 5 minutes, at 37°C, with 40 μl/cm^2 ^trypsin/EDTA (Gibco/BRL). Cells were harvested in a 10 fold greater volume of supplemented culture medium and enzyme was washed away by centrifugation at 140 × g for 3 minutes. The pellet was suspended in the supplemented culture medium and the cell suspension redistributed in collagen coated culture flasks. Samples from both cell culture types (colonocytes or jejunocytes) were frozen and stored in liquid nitrogen, at 1.5 10^6 ^cells per vial, in 1 ml of D-MEM containing 10 % DMSO (Merck) and 10 % FBS (Hyclone Perbio Sciences). We checked that frozen cells were suitable to reconstitute a culture.

### Cell viability

Viability of freshly isolated material was tested with a mixture of 10 mg/ml ethidium bromide (Sigma) and 5 mg/ml acridin orange (Sigma) in PBS. The mixture was added 1/1000 to the cell suspension and immediately observed using a fluorescence microscope (Nikon Eclipse TE 200). Under these conditions dead cells nuclei were stained in red while nuclei of living cells corresponded to green spots.

### Immunocytochemistry

Several antibodies were used to characterize the primary cell cultures, namely: monoclonal anti-pan-cytokeratin (mixture of clones C-11, PCK-26, CY-90, KS1A3, M20, A53 and B/A2, Sigma), monoclonal anti-E-cadherin (Becton Dickinson Biosciences), monoclonal anti-α smooth muscle actin (clone 1A4, Sigma), monoclonal anti-vimentin (clone V9, Sigma), monoclonal anti-epithelium specific antigen (ESA, Sigma) and monoclonal anti-cytokeratin peptide 18 (clone KS-BA2, Sigma). Prior to immunostaining, cells were fixed with 5% paraformaldehyde into PBS for 5 minutes at room temperature. Cells were then permeabilized by incubation for 15 minutes with 0.2% Triton X-100 (Roche) dissolved in PBS containing 5% goat serum (Gibco/BRL) to block non-specific binding. After 3 washes in PBS, for 5 minutes each, cells were incubated for 60 minutes at room temperature with the primary antibody in the blocking solution. Dilutions used were those recommended by the manufacturer. Cells were washed three times, for 5 minute each, in PBS and then incubated with the secondary antibody for 30 minutes at room temperature in darkness. The secondary antibody was anti-mouse IgG conjugated to FITC (Sigma) or TRITC to detect the antigen-antibody complex. After another three washes in PBS, cells were observed with a fluorescent microscope (Nikon Eclipse TE 200). In a few cases, direct immunofluorescence was performed using an anti-pan-cytokeratin (clone C11) FITC conjugate (Sigma) and anti-vimentin (clone V9) Cy3 conjugate (Sigma). For epithelia specific staining, Caco-2 cells (human carcinoma colonic cells, received from Prof. YJ Schneider, Catholic University of Louvain, Belgium) were used as positive controls. For all other immunodetections, 3T3 fibroblasts (a generous gift from Prof. E. Heinen, Histology Laboratory, University of Liege, Belgium) and bovine cell samples submitted to the staining procedure, but omitting the incubation with primary antibody, were used as negative controls.

### Western Blot

Samples of fresh mucosa and cell suspension used for starting the cultures were prepared by washing the cells twice into PBS and suspending the pellet in ultrapure water (1 ml). Samples of confluent cultured cells were prepared by discarding the culture medium, rinsing the cell monolayer twice with PBS and then cell scraping in ultrapure water (300 μl ultrapure water for a 25 cm^2 ^culture flask). Samples were sonicated (30 sec on ice, Sonic Power Company Cell Disrupter) to accomplish cell lysis. Twenty μg of proteins from each sample were separated by electrophoresis on 10% SDS-PAGE [50] and transferred to Bio Trade PVDF Transfer Membrane (0.45 μm, Pall Corporation, Life Sciences). After 1 hour incubation at 37°C in the blocking solution (PBS containing 0.2% of Tween 20 and 5% of milk powder; Nestle), membranes were incubated for 1 hour at 37°C (or overnight at 4°C) with primary antibody (see immunocytochemistry section) diluted in the blocking solution according the manufacturer's instruction. Three washes in the blocking solution were followed by the secondary antibody incubation. The secondary antibody was a peroxydase conjugate (Antimouse IgG peroxydase conjugate; Sigma) diluted at 1:1000 in the blocking solution. The blots were developed using the peroxydase substrate 3,3-diaminobenzidine (DAB; Sigma) containing 0.03% H_2_O_2 _(Merck). Cultured Caco-2 cells and 3T3 fibroblasts, submitted to the same treatment, were respectively used as positive and negative controls for epithelial immunostaining.

### Gene expression analysis

Gene expression analyses of cell samples were realized using the combined mRNA reverse transcription-polymerase chain reaction (RT-PCR). Total RNA was extracted using the guadinium thiocyanate protocol [51]. Prior to reverse transcription RNA was treated with DNAse (DNAse RQ1; Promega) in the appropriate DNAse buffer (RQ1 Buffer; Promega) for 30 minutes at 37°C to break up the possible contaminant DNA. Reverse transcription of mRNA was performed from 2 μg of total RNA, in presence of RNAse inhibitor (RNAguard 40 U/μl; Promega) using oligo-dT primers (Oligo dT-15 Primer, Promega), deoxynucleotides (dNTP 10 mM, Promega), Mo-MuLV reverse transcriptase (200 U/μl, Promega) and the reverse transcriptase buffer (Promega) in a 150 μl final volume. Reverse transcription was performed at 42°C for 1 h. Primers used for the specific amplification of cDNA fragments corresponding to coding segments of the following proteins were: villin forward 5'-ACC-TTC-ACA-GGC-TGG-TTC-CT-3' and reverse 5'-GGT-TTT-GTT-GCT-TCC-AT-3' (amplification product size: 384 bp); intestinal peptidase (IP): forward 5'-GCG-ATT-ATG-CCC-CTT-TCA-TT-3' and reverse 5'-CAG-CCT-GCA-GGA-AGC-T-3' (amplification product size: 276 bp); Fatty Acids Binding Protein (FABP) forward: 5'-TTC-AGC-AGC-TGG-TAG-GAA-3' and reverse 5'-TAA-CCA-AAG-AGA-TGA-CCC-TA-3' (amplification product size: 276 bp); Zonula Occludens 1 (ZO1) forward: 5'-GCG CTG AAA GAA GCA ATT CA-3' and reverse: 5'-AAA CAT GGT TCT GCC TCA TC-3' (amplification product size: 272 bp); vimentin: forward 5'-CCG-GAG-CTA-CGT-GAC-CAC-AT-3' and reverse 5'-CTC-GGC-TTC-CTC-TCT-CTG-AA-3' (amplification product size: 540 bp); E-cadherin: forward 5'-CGC-ACA-ACA-AAA-TGT-TCA-CC-3' and reverse 5'-CAT-TGG-TGA-CTG-GGT-CTG-TG-3' (amplification product size: 308 bp). RNA extraction and RT-PCR success were checked through the co-detection of the constantly expressed gene β-actin, using the primer pair forward: 5'-AGA-AAA-TCT-GGC-ACC-ACA-CC-3' and reverse: 5'-GTC-AGG-CAG-CTC-GTA-GCT-CT-3' (amplification product size 485 bp). PCR comprised 35 cycles, each cycle consisting in the succession of a denaturing step at 95°C for 1 minute, a primer annealing step (at 50 to 55°C, depending on the primer pair) for 1 minute and a primer elongation step 72°C for 1 minute. They were preceded by a first denaturing step for 3 minutes at 95°C and followed by a final elongation step at 72°C for 5 minutes. The PCR reaction contained 1 to 2 μl of cDNA, 1 μmol/L of each primer (Eurogentec, Belgium), 1.5 mmol/L of MgCl_2 _(Promega), 200 μmol/L of deoxynucleotide triphosphates (dNTP 10 mM, Promega) and 0.5 units of Taq polymerase (Promega) in the appropriate buffer (Promega). Control reactions omitting the cDNA were included and yielded negative response.

Amplification products were visualized after electrophoresis on 1% agarose (Sigma) gels in Tris Borate EDTA buffer pH 8 (TBE; 89 mM Tris base, Sigma; 89 mM boric acid, Merck; 2 mM Na EDTA, Merck) stained with ethidium bromide (1‰ w/v, Sigma). Samples were dissolved in electrophoresis loading buffer (Promega). Simultaneous migration of DNA molecular weight standards (1 Kb ladder, Promega) allowed the size determination (in bp) of amplification products.

### Enzymatic activities

Samples of fresh mucosa, cell suspension used to start the cultures and epithelial cell cultures were prepared as described for western blot analysis. Maltase activity (brush border disaccharidase) was measured according to the spectrophotometric method developed by Dahlqvist [52]. Alkaline phosphatase was estimated using 4-nitrophenyl phosphate as substrate (1.5 mM; Acros Organics) in ethyldiethanolamine buffer (150 mM; Sigma) and dinitrophenolphosphate (Sigma) as standard. After a 20 minute incubation time, the reaction was stopped by a 1 M NaOH solution. The absorbance of the enzyme reaction product was measured at 415 nm. Enzymatic activities were reported to the protein content measured by the method of Bradford [53] and expressed as specific enzymatic activities (SA, μmol/min.g).

### Electron microscopy

For transmission electron microscopy, cells were grown in collagen I coated culture wells. Cultured cells were washed twice with PBS and then fixed in 2.5% glutaraldehyde solution at 4°C for 1 hour. Two other PBS washes were followed by a post-fixation in 2% osmium tetroxyde for 1.5 h. Then samples were dehydrated in serial ethanol dilutions, scrapped from their plastic support and embedded in epon-epoxy resin. Ultrathin sections were counterstained with uranyl acetate were fixed on grids and observed under a transmission electron microscope (JEOL JEM-100CX) at 80 kV.

### Statistics

Results are reported as mean ± standard deviation (SD). With regard to heteroscedasticity, statistical analysis was performed using Newman-Student-Keuls or Kruskal-Wallis ANOVA. A p-value below 0.05 was considered as statistically significant.

## Authors' contributions

DR carried out all the experimental analysis, participated to the design of the study and to the manuscript drafting. SL designed the study, initial culture protocols and molecular biology methods and supervised the first author in the paper writing with a major contribution to the discussion section. OP performed the statistical analysis, realized the artwork and helped to draft the manuscript. JM contributed to the design of the study and helped the access to animal tissues used in this study. GD participated to the design of the study and helped to draft the manuscript. All authors read and approved the final manuscript.
